# Monogamy in a Hyper-Symbiotic Shrimp

**DOI:** 10.1371/journal.pone.0149797

**Published:** 2016-03-02

**Authors:** J. Antonio Baeza, Lunden Simpson, Louis J. Ambrosio, Rodrigo Guéron, Nathalia Mora

**Affiliations:** 1 Department of Biological Sciences, 132 Long Hall, Clemson University, Clemson, SC 29634, United States of America; 2 Smithsonian Marine Station at Fort Pierce, 701 Seaway Drive, Fort Pierce, Florida 34949, United States of America; 3 Departamento de Biología Marina, Facultad de Ciencias del Mar, Universidad Católica del Norte, Larrondo 1281, Coquimbo, Chile; 4 Instituto Federal de Educação, Ciência e Tecnologia do Espírito Santo - Campus Alegre, Espírito Santo, Brasil; 5 Departamento de Biología, Facultad de Ciencias, Universidad del Valle, Cali 760032, Colombia; University of Natural Resources and Life Sciences, Vienna, AUSTRIA

## Abstract

Theory predicts that monogamy is adaptive in resource-specialist symbiotic crustaceans inhabiting relatively small and morphologically simple hosts in tropical environments where predation risk away from hosts is high. We tested this prediction in *Pontonia manningi*, a hyper-symbiotic shrimp that dwells in the mantle cavity of the Atlantic winged oyster *Pteria colymbus* that, in turn, infects gorgonians from the genus *Pseudopterogorgia* in the Caribbean Sea. In agreement with theory, *P*. *manningi* were found dwelling as heterosexual pairs in oysters more frequently than expected by chance alone. Males and females also inhabited the same host individual independent of the female gravid condition or of the developmental stage of brooded embryos. While the observations above argue in favor of monogamy in *P*. *manningi*, there is evidence to suggest that males of the studied species are moderately promiscuous. That females found living solitary in oysters most often brooded embryos, and that males allocated more to weaponry (major claw size) than females at any given size suggest that males might be roaming among host individuals in search of and, fighting for, receptive females. All available information depicts a rather complex mating system in *P*. *manningi*: primarily monogamous but with moderately promiscuous males.

## Introduction

**Fig 1:**
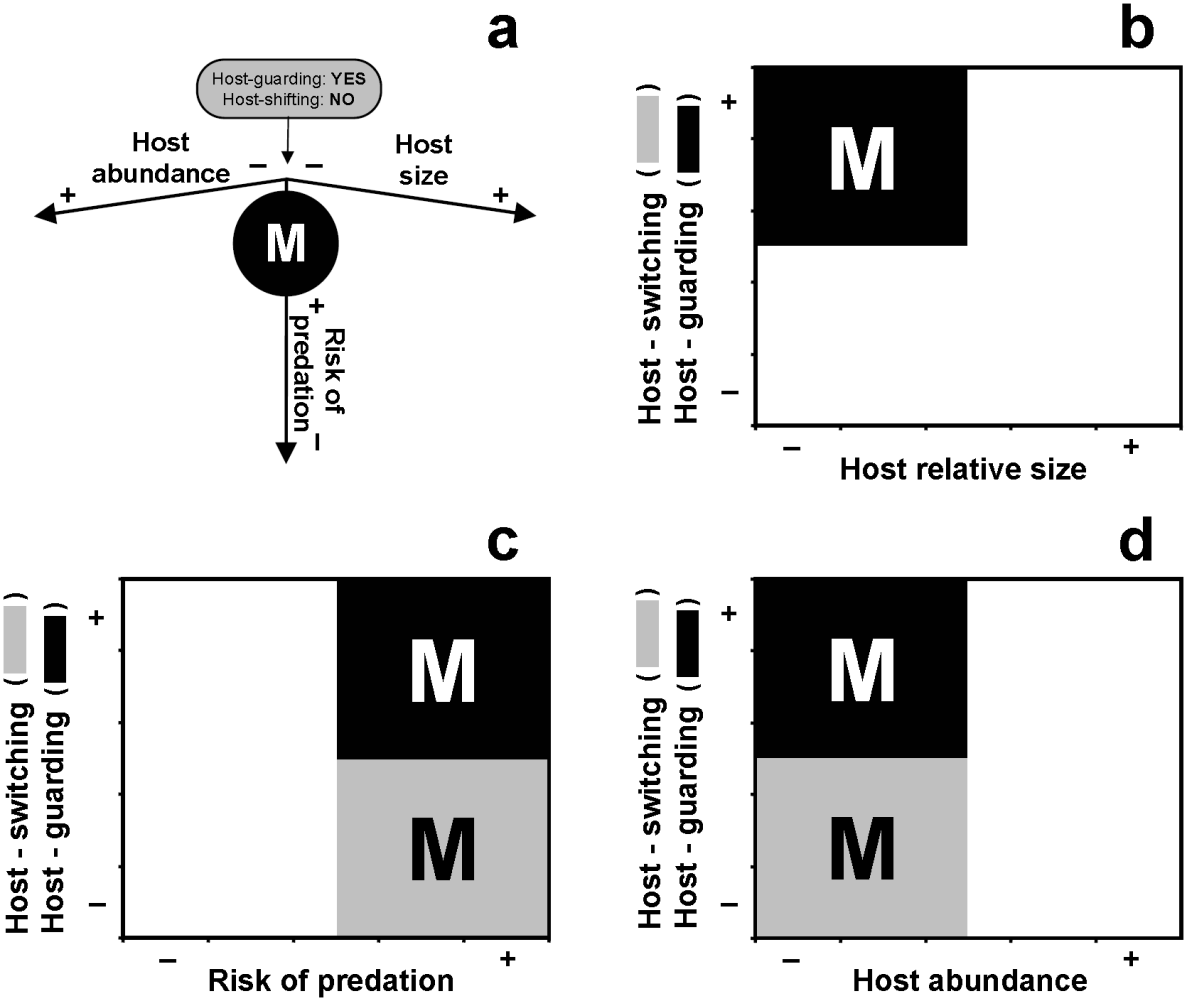
The adaptive value of monogamy. Monogamy is predicted for resource-specialist symbiotic crustaceans according to environmental, including host, conditions (*sensu* model formulated in [11]). (a) The interaction among predation pressure, host relative size, and abundance is envisioned as a tri-dimensional landscape on which different mating systems occur. Theory predicts that monogamy is adaptive in symbiotic crustaceans inhabiting relatively small and morphologically simple hosts in tropical environments where predation risk away from hosts is high. Movement among hosts (i.e., host-switching) is constrained and monopolization of hosts (host-guarding) is favored in males and females due to host scarcity and its value in offering protection. Given that host spatial constraints allow only a few adult symbiotic individuals to cohabit the same host, both sexes are expected to maximize their reproductive success by sharing 'their' dwelling (i.e., host individual) with a member of the opposite sex. Symbiotic guest individuals are typically rejected from hosts by members of the same sex, but not by members of the opposite sex—a behavior eventually leading to social monogamy and long-lasting heterosexual pairing [11]. (b), (c), and (d) The different decisions on host-monopolization and host-switching (i.e., among-host individual movements) expected by symbiotic crustaceans depending upon host characteristics and predation risk away from host individuals according to an optimality economical approach. (b) Host-guarding is favored when host body size (relative to that of symbiotic guests) is small. Host-switching is neither favored nor constrained by host body size. For further details, see text and [11]. (c) Host-guarding is favored when predation risk away from hosts is high. Host-switching is favored when predation risk away from hosts is low and constrained when predation risk away from hosts is high. (d) Host-guarding is favored when host abundance is low. Host-switching is favored when host abundance is high and constrained when host abundance is low [11]. In (a), (b), (c), and (d), the M within circles or squares indicate when monogamy is favored by environmental conditions.

Social monogamy (herein defined *sensu* [1] as pairs of conspecifics spending extensive periods of time together) has evolved multiple independent times in marine and terrestrial environments among invertebrate and vertebrate organisms that may or may not exhibit parental care (e.g., the snapping shrimp *Alpheus armatus* [2]; the California mouse, *Peromyscus californicus* [3]; the Lake Tanganyika cichlid fish *Tropheus moorii* [4]; the human *Homo sapiens* [5]; among many others). In species with biparental care, the benefits arising from shared parental duties when rearing expensive offspring (both in terms of energy and time) appear to explain its adaptive value [6–7]. In the absence of biparental care, various other hypotheses have been put forward to explain the adaptive significance of monogamy (e.g., “territorial cooperation” hypothesis [8]; “mate-guarding” hypothesis [9–10]; among others). Most recently, Baeza and Thiel [11] proposed that social monogamy is advantageous in refuge-specialist organisms inhabiting environments in which refuges are small and support few (e.g., two) individuals, when these refuges are scarce and when predation risk away from refuges is high (see also [12–14]). Under the conditions above, movement among refuges is hindered and their monopolization is favored due to refuge scarcity as well as their large value in offering protection against predators [11]. Because spatial limitation allows only a few reproductive individuals to cohabit the same refuge, both males and females are expected to maximize their reproductive success by sharing 'their' dwelling with a member of the opposite sex [11, 15] (Fig 1).

Taking into account the above, social monogamy should be adaptive in numerous symbiotic organisms from disparate phylogenetic origins that inhabit tropical environments (symbiosis here defined *sensu* [16] as dissimilar organisms living together). Low tropical latitudes are characterized by the large diversity of scarce biotic (e.g., benthic macro-invertebrates: sponges, corals, annelids, echinoderms and tunicates, among many others) refuges (i.e.. symbiotic hosts) that are used as a shelter, food source, nursery ground and/or mating arena by a wide variety of small resource-specialist (i.e., symbiotic guest) organisms [11]. In agreement with the above notion, various studies conducted during the last decade have shown that symbiotic guest species inhabiting scarce, small, and structurally simple host species in reef environments, in which mortality risks for symbiotic guests when away from hosts is high, are socially monogamous [2, 13, 17–19]. On the other hand, a few studies have also found non-monogamous and putatively promiscuous symbiotic species inhabiting environments that should favour monogamy (e.g., *Ascidonia flavomaculata* [19]; *Odontonia katoi* [20]). Certainly, more studies on the biology of resource-specialists, including symbiotic guest species, are needed to continue improving our understanding of those conditions favouring social monogamy in species without parental care.

In this study, we test the hypothesis stating that symbiotic organisms living in association with small, simple, and sparse host species in habitats where the risk of mortality away from host individuals is high, exhibit a socially monogamous mating system [11]. We used the caridean shrimp *Pontonia manningi* as a model system. *Pontonia manningi* is a hyper-symbiotic species that dwells in the mantle cavity of *Pteria colymbus*, the Atlantic winged oyster, which in turn attaches to living colonies of various gorgonian corals from the genus *Pseudopterogorgia* (and *Leptogorgia*) in the Caribbean Sea [21–22]. The hyper-symbiotic lifestyle of *P*. *manningi* likely implies low host availability for this shrimp given that locating a suitable host is dependent on and limited by the availability of a host species that requires a specific host itself. The winged oyster *Pteria colymbus*, used as a host by this shrimp, represents a small refuge (under 20 mm in shell length [21]) that should be easy to protect and defend against intruders [23, 13]. It is highly likely that movement among oysters in shallow subtidal reefs is costly for both male and female shrimp because of the risk of predation by the omnivorous fishes and crabs common in these species-rich tropical reef environments [24–25]. Environmental constraints such as the above that limit the ability of symbiotic individuals from the two sexes to switch among the relatively small and scarce hosts in search of sexual partners should favor social monogamy in symbiotic associates [11, 20].

A sound approach to test whether or not social monogamy is adaptive in symbiotic species, including *P*. *manningi*, inhabiting environments expected to favour such a mating system, is to describe the population distribution of symbiotic species and to tag and track the fate of symbiotic individuals [26]. Unfortunately, given the cryptic nature of many symbiotic, including hyper-symbiotic, species, direct long-term observations on the activity and among-host movement of symbiotic individuals are rarely plausible [13–14, 27] (for one of a few exceptions [26]). Nonetheless, various recent studies have demonstrated the possibility of inferring the mating system of symbiotic species after detailed examination of their population distribution, male–female association pattern, host–shrimp body size relationship, and sexual dimorphism in terms of body size and weaponry [2, 11, 13, 20, 26, 28–31].

Specifically, if *P*. *manningi* is socially monogamous, then it is expected that [i] the population distribution of this shrimp in *Pteria colymbus* is non-random with paired shrimps found more often than expected by chance alone, and [ii] the sex distribution of crabs in pairs is non-random with male-female pairs being found more often than expected by chance alone. [i] and [ii] above represent strong evidence that *P*. *manningi* shrimps actively choose to share hosts individuals with members of the opposite sex [rather than to live solitarily or in aggregations] as expected to occur in monogamous species [11]. Furthermore, if *P*. *manningi* is monogamous, then, it is expected that [iii] males pair with females regardless of their reproductive state (e.g., the presence / absence of eggs and egg developmental stage), [iv] male-female pairs display size-assortative pairing and [v] shrimp body size is positively correlated with host body size. [iii], [iv], and [v] above argue in favour of long-term stability of paired shrimps; heterosexual pairs grow together under similar space- and resource-related constraints for long periods of time [13, 18, 29]. Lastly, [vi] *P*. *manningi* should display little to no sexual dimorphism in body size and weaponry (e.g., chelipeds used for intra-sexual aggression) [11–12]. The low intensity of sexual selection characteristic of monogamous regimes is expected to relax selection for large body size and weapons in males [11, 13, 15].

Here, we describe the population distribution, male-female association pattern, and sexual dimorphism of *P*. *manningi* to gain better insight into the adaptive value of social and mating strategies in resource-specialists.

## Material and Methods

### Collection of hosts and shrimps

**Fig 2:**
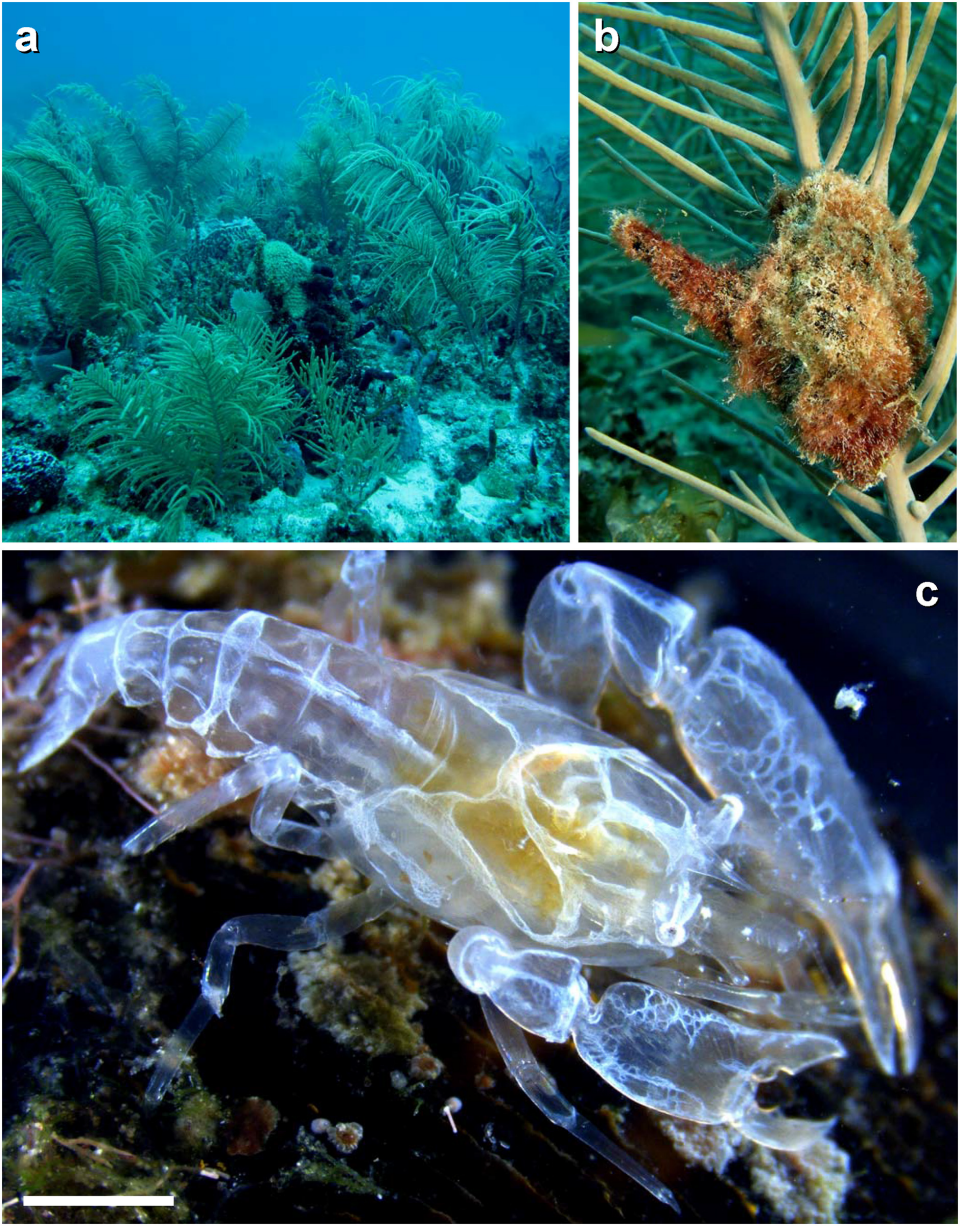
Sampling sites and symbiotic model organisms. (a) Shallow subtidal at one of the study sites, FLKReef3, Long Key Reef track, Florida Keys, Florida, USA. (b) Solitary individual of the Atlantic winged oyster *Pteria colymbus* attached to a host colony of *Pseudopterogorgia* sp. (c) A male of *Pontonia manningi* (scale bar = 1.6 mm). Photo credits: J. Antonio Baeza

Florida Wildlife Commission gently granted permission for invertebrate collection. Specimens of the Atlantic winged oyster *Pteria colymbus* were collected between June 22nd and 25th, 2015 using SCUBA from the shallow subtidal (7–12 m) at 3 different sampling sites, named FLKReef1, FLKReef2, and FLKReef3, all located in the Florida Reef tract, approximately 4.8 km off Long Key (24.8190° N, 80.8140° W), Florida Keys, Florida, USA. The different sampling sites were low relief coral reefs dominated by soft corals (i.e., *Pseudopterogorgia* spp.) but also exhibiting a highly diverse array of sessile macroinvertebrates (Fig 2a), including several sponge species, i.e., the vase sponge *Callyspongia vaginalis* and the barrel sponge *Xetospongia muta*. Individuals of *P*. *colymbus* were found either solitarily or in pairs (see results) attached to the stems of their gorgonian host corals by byssus threads (Fig 2b). Species of predatory fishes (known to prey upon crustaceans [24–25]) were observed at all study localities, including various species of search-and-catch (e.g., wrasses *Halichoeres* spp.) and sit-and-wait fish predators (the invasive lionfish *Pterois volitans*, the scorpionfish *Scorpaena* spp.). Also, often observed at the sampling sites were various omnivorous / predatory damselfish from the genus *Abudefbuf* and *Chromis*, and unidentified gobies and blennies.

To determine the prevalence and intensity of the association between *P*. *colymbus* and their soft coral hosts, we searched for the first 30 *Pseudopterogorgia* spp. colonies upon starting our dives at each site. We did not sample *Leptogorgia* spp. during this study given the low abundance of this soft coral at our sampling sites. The size (maximum height, cm) of each of these first 30 coral colonies was measured with a ruler (precision = 0.1 cm) and the presence/absence of *P*. *colymbus*, and number of oysters found on each colony was recorded. If *P*. *colymbus* oysters were found, they were removed from the soft coral and placed in individually tagged resealable plastic bags. After sampling the first 30 gorgonians found, we then haphazardly collected additional oysters (50 < N_total_ < 70) at each study site. Each collected oyster was also individually placed in a resealable plastic bag and transported to the laboratory in Long Key, Florida. In the laboratory, oysters were gently dissected with a scalpel, the number of shrimp per oyster was recorded, and each shrimp found in the mantle cavity was fixed in 95% Ethanol for transportation to a second laboratory in Clemson University, South Carolina. Lastly, the maximum length of the left or right valve of each winged oyster was measured with a calliper to the nearest 0.1 mm.

### Host use pattern of *Pontonia manningi*

To test for monogamy in *P*. *manningi*, we examined the host use pattern of this shrimp, which includes a description of its population distribution, male–female association pattern and host–shrimp body size relationships. First, we examined whether or not symbiotic shrimps live solitarily, in aggregations or in pairs within the mantle cavity of the winged oyster. For this purpose, we examined whether or not the distribution of *P*. *manningi* in the oyster host (i.e., the frequency of occurrence of hosts without shrimps and with different numbers of shrimps) differed from a random distribution. We compared the observed distribution (i.e., frequency of occurrence of hosts with zero, one, two, three or more shrimps) with the Poisson random distribution [32]. Significant differences between the distributions were examined using a Chi–square test of goodness–of–fit [33]. When significant differences were observed, specific frequencies between the observed and expected distributions were compared by subdivision of the Chi–square test and using the sequential Bonferroni correction to control for false discovery rate [34].

A relatively large proportion of oyster hosts were found to contain pairs of shrimps (see results). To determine whether the sexes were randomly distributed among shrimp pairs inhabiting the same host, the observed distribution was compared with the binomial distribution. The expected random frequencies of distribution of the different sexes were calculated based on the proportion of males and females recorded in the population. A Chi-square test of goodness–of–fit was used to inspect for significant differences between the distributions as indicated above [33].

### Sexual dimorphism in *Pontonia manningi*

In caridean shrimps from the family Palaemonidae, including representatives from the genus *Pontonia*, the second pair of thoracic appendages bears the larger of the two pair of claws [23, 35]. In shrimps, these structures serve as weapons during intra-sexual interactions or for inter-sexual communication [35]. In turn, the left and right pleura of the second abdominal segment are greatly enlarged and help protect the embryos (i.e., from physical abrasion) carried by females beneath their abdomen [35].

We examined whether the largest cheliped on the second pair of pereopods and the left pleuron of the second abdominal segment increase linearly with body size in males and females of *P*. *manningi* following [13]. In short, the relationship between the length of the propodus of the largest second cheliped or the length of the pleuron of the second abdominal segment and body size of shrimp (CL, mm) was examined using the allometric model y = ax^b^ [36, 37]. The slope b of the log-log least-squares linear regression represents the rate of exponential increase (b>1) or decrease (b<1) of the cheliped and abdominal segment with a unit of increase in body size of shrimp. To determine if the relationship deviates from linearity, a t-test was used to test if the estimated slope *b* deviates from the expected slope of unity [38]. If the cheliped or the abdominal pleuron grows more or less than proportionally with a unit increase in body size of shrimp, then the slope should be greater or smaller than the unity, respectively [36].

## Results

### Host use pattern of *Pontonia manningi*

Between 52 and 61 individuals of the Atlantic winged oyster *Pteria colymbus* were collected from *Pseudopterogorgia* spp. at the three different sampling sites in the Florida Reef tract, Florida Keys (Table 1). The average (± S.D) density of the soft coral *P*. spp., host to *Pteria colymbus*, at the study sites varied between 1.31 (± 0.03) m^2^ and 2.05 (± 0.27) m^2^ in FLKReef1 and in FLKReef3, respectively. Prevalence of the oyster *P*. *colymbus* in the soft coral *Pseudopterogorgia* spp. varied between 6.7% and 21.3% in FLKReef3 and in FLKReef1, respectively. Considering only those soft corals harboring *Pteria colymbus*, the numbers of oysters per host soft coral (oyster intensity) varied between 1 (± 0) and 2.38 (± 1.71) in FLKReef3 and FLKReef1, respectively. *Pteria colymbus* was most often found solitarily (56.5% of the soft corals harboring one winged oyster) in host corals or in clusters comprised of 2 to 4 individuals (39.1% of the soft corals), and only once in a large group composed of 7 individuals.

**Table 1:** Host-use pattern of *Pteria colymbus* (PC) on *Pseudopterogorgia* spp. (Psp.), and host-use pattern of *Pontonia manningi* (PM) in *Pteria colymbus*.

	Reef		
	FLKReef1	FLKReef2	FLKReef3
Psp. Density (ind./m^2^)	1.31 ± 0.03	1.5 ± 0.28	2.05 ± 0.27
	(63–67)	(62–91)	(90–117)
PC Prevalence (%)	21.3%	18.6%	6.7%
	59	59	59
PC Density (ind./coral)	0.53 ± 1.26	0.24 ± 0.63	0.07 ± 0.25
	(0–7)	(0–4)	(0–1)
PC Intensity (ind./coral)	2.38 ± 1.71	1.50 ± 1.22	1 ± 0
	(1–7)	(1–4)	(1–1)
PC Length (OL, mm)	56.31 ± 12.26	56.01 ± 9.89	54.32 ± 11.45
	(25.9–82.75)	(15.1–81.25)	(22.5–78.3)
PM Prevalence (%)	19.2%	39.1%	19.7%
	52	64	61
PM Density (ind./oyster)	0.35 ± 0.74	0.67 ± 0.87	0.36 ± 0.72
	(0–2)	(0–2)	(0–2)
PM Intensity (ind./oyster)	1.80 ± 0.42	1.64 ± 0.49	1.67 ± 0.49
	(1–2)	(1–2)	(1–2)
PM CL (mm)	4.28 ± 0.92	3.47 ± 0.76	3.35 ± 0.54
	2.86–6.18	1.8–5.1	2.61–4.86

Measurements taken from three different sampling sites are represented as FLKReef1, FLKReef2, and FLKReef3 (Florida Long Key Reef 1, Florida Long Key Reef 2, and Florida Long Key Reef 3, respectively). Mean ± SD (range) for all measurements except oyster and shrimp prevalence (%) which report % and total N of corals and oysters sampled.

The average (± S.D) length of the sampled oysters varied between 56.31 (± 12.26) mm and 54.32 (± 11.45) mm at FLKReef1 and FLKReef3, respectively (Table 1). The population size distribution of the oysters was similar among the three sampling sites and there was no significant difference in oyster average shell length among localities (ANOVA; F = 0.51, df = 2,166, *P* = 0.6024).

**Fig 3:**
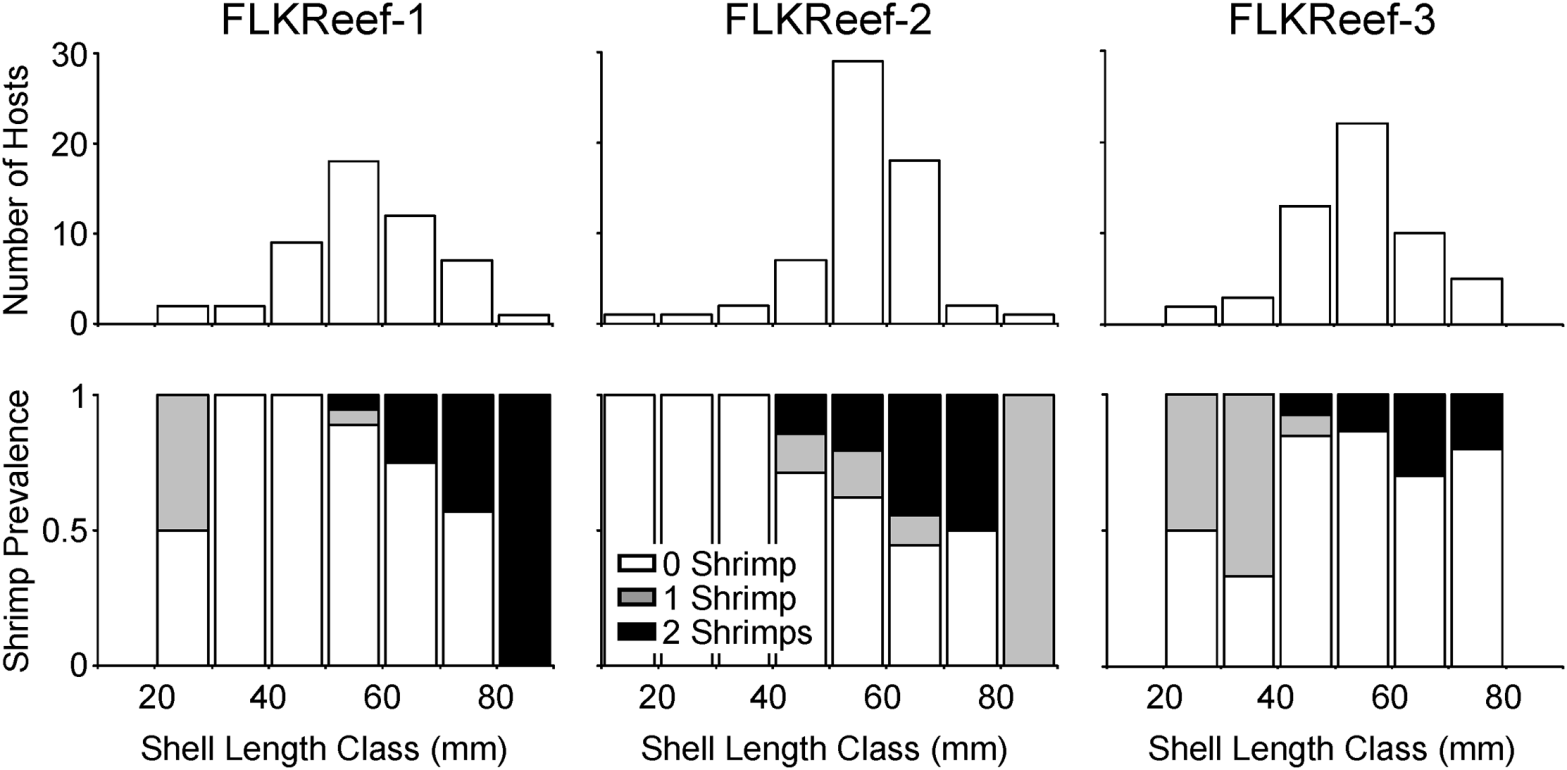
Size-frequency distribution of the Atlantic winged oyster *Pteria colymbus* and frequency of occurrence of *Pontonia manningi* shrimps in host individuals. (top) Size-frequency distribution of the Atlantic winged oyster *Pteria colymbus* at the three different sampling sites in the Long Key Reef track. (bottom) Frequency of occurrence of *Pontonia manningi* shrimps in oyster host individuals of different size classes at the three different sampling sites in the Long Key Reef track (see Results for further details).

Prevalence of *Pontonia manningi* in *Pteria colymbus* varied between 19.2% and 39.1% in FLKReef1 and FLKReef2, respectively (Table 1). The difference in the frequency of occurrence of shrimp in oysters among the different study localities was significant (χ^2^ test of independence; χ^2^ = 8.32, df = 2, *P* = 0.016). Prevalence of *Pontonia manningi* in *Pteria colymbus* was greater at FLKReef2 compared to FLKReef1 and FLKReef3 (a-posteriori decomposition of χ^2^ test of independence; FLKReef2 vs FLKReef1 + FLKReef3: χ^2^ = 8.25, df = 1, *P* = 0.004). At FLKReef1 and FLKReef2, most oysters with shrimp were large (> 50 mm SL). By contrast, at FLKReef3, oysters from all body sizes harbored shrimp with similar frequencies (Fig 3). However, only medium and large body size oysters harbored more than one shrimp at FLKReef3 (see below).

**Fig 4:**
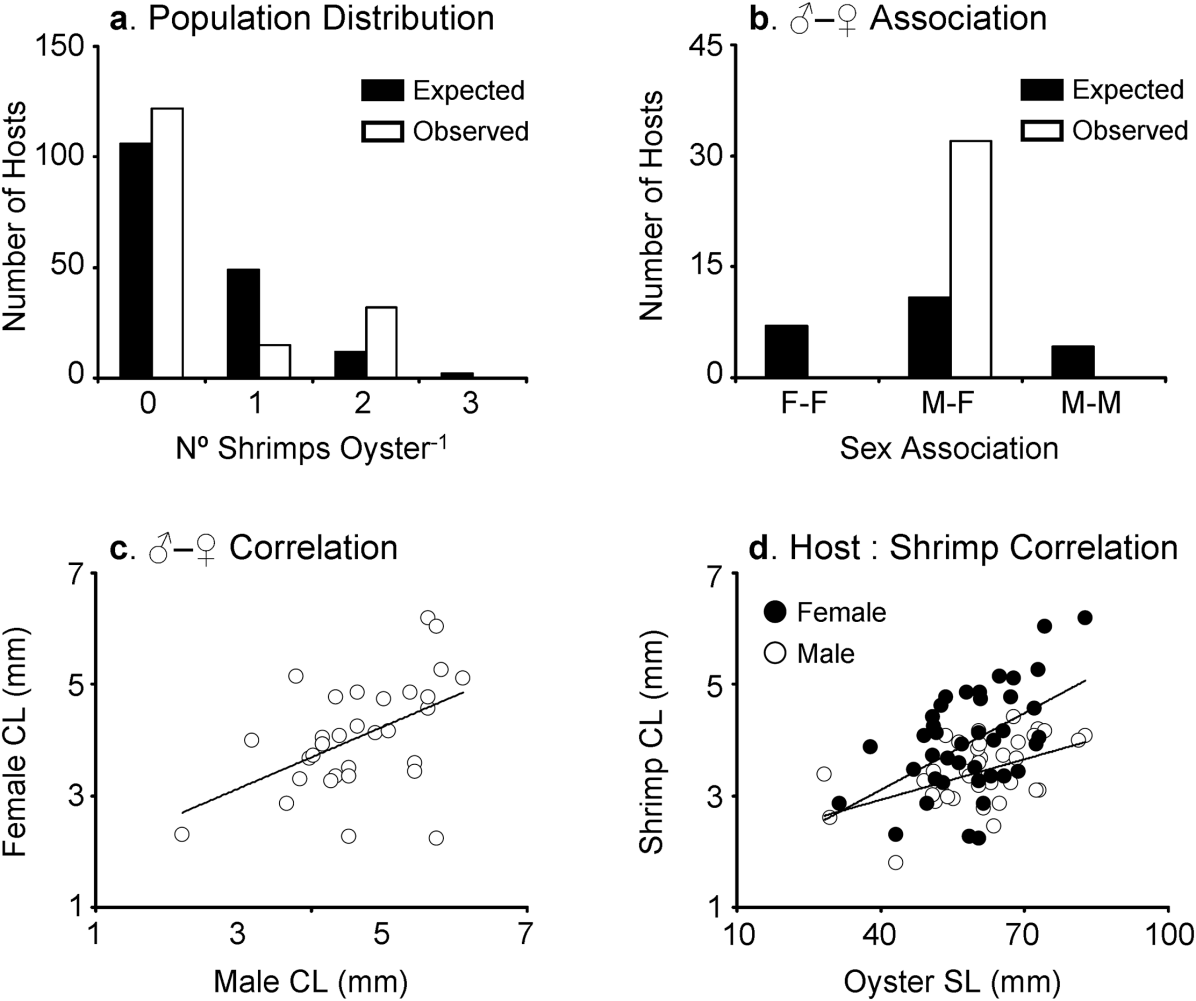
Host use pattern of *Pontonia manningi* (combined data from the three sampling sites) at Long Key Reef tract, Florida Keys, USA. (a) Population distribution of *Pontonia manningi*. Observed frequency of shrimps on hosts differed significantly from an expected Poisson random distribution. (b) Male-female association pattern of *Pontonia manningi* found as pairs inside the mantle cavity of the Atlantic winged oyster *Pteria colymbus*. Observed frequency of heterosexual pairs differed significantly from the expected binomial random distribution. (c) Relationship between carapace length (CL) of males and females of *Pontonia manningi* found as pairs inside the mantle cavity of the Atlantic winged oyster *Pteria colymbus*. (d) Relationship between carapace length (CL) of males or females of *Pontonia manningi* and shell length of the Atlantic winged oyster *Pteria colymbus*. See results for further details.

For the analysis of population distribution and male-female association pattern in *P*. *manningi*, the data from all localities were pooled together because of the absence of differences among sampling sites in the body size of hosts and the minimal differences in occupancy pattern of hosts by shrimp. A total of 38 males and 38 females were retrieved from oysters in all study sites. The sex ratio was unbiased in the population (sex ratio = 0.5; Fisher's Exact test, P = 0.0). The density of *P*. *manningi* on oysters (number of shrimp per host calculated including oysters where no shrimp was found) varied between 0 and 2 with a mean of 0.47 ± 0.79. In turn, the intensity of *P*. *manningi* on oysters (number of shrimp per host calculated considering oysters where one or more shrimp were found) varied between 1 and 2 with a mean of 1.68 ± 0.47. The population distribution of *P*. *manningi* on oysters did not display a random pattern (Chi-square test of goodness–of–fit, χ^2^_2_ = 16.12, *P* = 0.0003). This was explained by the larger number of oysters harboring two shrimp compared to the number expected by chance alone (decomposition of the Chi-square test of goodness–of–fit; χ^2^_1_ = 7.83, *P* = 0.0051, sequential Bonferroni α = 0.025) and due to the smaller number of oysters harbouring one shrimp compared to the number expected by chance alone (χ^2^_1_ = 32.25, *P* < 0.0001, sequential Bonferroni α = 0.05) (Fig 4a).

A total of 14 oysters harbored a single shrimp; 7 males and 7 females (4 of which were brooding embryos). A total of 32 oysters harbored two shrimps. Pairs of shrimp inhabiting oysters invariably consisted of one male and one female (20 of which were brooding embryos: stage I embryos = 1, stage II embryos = 8, stage III embryos = 2, stage IV embryos = 9). No oyster hosts harbored two male or two female shrimps (Fig 4b). Taking into consideration the binomial distribution and the relative abundance of males and females in the studied populations, the number of hosts harbouring heterosexual pairs expected by chance alone would have been 16. Therefore, paired shrimp were found to be heterosexual more frequently than expected by chance alone (Fig 4b).

There was a positive correlation between the size (CL) of males and females found as pairs (t-test; t_1,30_ = 3.08, *P =* 0.0044) (Fig 4c); 23.99% of the variation in female size was explained by male size (r^2^ *=* 0.2399). In all but five of the heterosexual pairs, the female was larger than the male. Also, a positive correlation between the size of the major cheliped of males and females found as pairs was recorded (t-test; t_1,33_ = 7.88, *P =* 0.0084); 19.8% of the variation in female cheliped size was explained by male cheliped size (r^2^ *=* 0.1976).

Solitary and paired males were, respectively, 3.36 ± 0.51 mm CL and 3.49 ± 0.51 mm CL. Solitary and paired females were, respectively, 3.79 ± 0.64 mm CL and 4.12 ± 0.94 mm CL. A two-way ANOVA did not detect any effect of group size (solitary versus paired) in shrimp body size (*F* = 1.04, d.f. = 1,75, *P* = 0.3116). On the other hand, sex (male versus female) did affect shrimp body size (*F* = 6.02, d.f. = 1,75, *P* = 0.0166). The interaction term of the ANOVA was not significant (*F* = 0.18, d.f. = 1, 75, *P* = 0.6713).

There was a statistically significant correlation between host size and shrimp size, regardless of the presence or absence of other shrimp in the same host, for both males and females (t-test; t = 11.61, df = 1,36, *P* = 0.0016, and t = 3.67, df = 1,38, *P* = 0.0007, for males and females, respectively). Though the correlations were statistically significant, only 24.38% (r^2^ = 0.2438) and 26.15% (r^2^ = 0.2615) of the variation in male and female body size, respectively, was explained by host size (Fig 4d).

### Sexual dimorphism in *Pontonia manningi*

**Fig 5:**
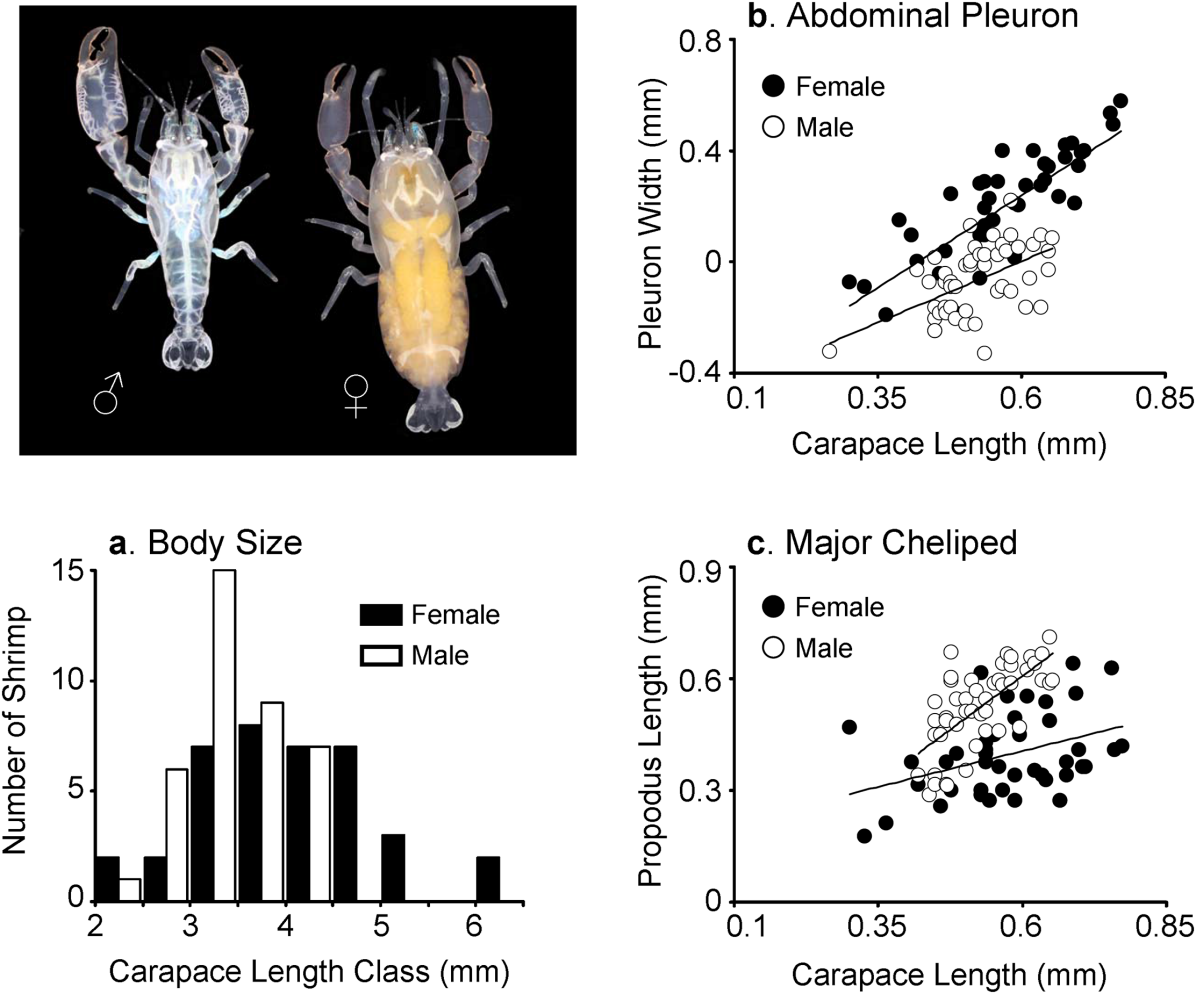
Sexual dimorphism in *Pontonia manningi*. (a) Size frequency distribution of body size (CL) in males and females. Measurements are in mm. (b) Relative growth of major cheliped propodus length as a function of carapace length in males and females of *Pontonia manningi*. (c) Relative growth of pleuron of second abdominal segment as a function of carapace length in males and females of *Pontonia manningi*. Linear regression equation obtained previous log-log transformation of the data are shown for each sex on Table 2. The photograph on the top left shows a male-female couple of *Pontonia manningi* retrieved from a single oyster.

The carapace length (CL) of male and female shrimp varied between 2.45 and 4.40 mm (mean ± SD, 3.46 ± 0.50) and between 2.24 and 6.10 mm (4.05 ± 0.89), respectively. The CL of females was larger than that of males (Kruskal-Wallis test [variances were not homogeneous]; S = 1151, Z = -3.24, P = 0.0012) indicating sexual dimorphism (males < females) with respect to body size in *P*. *manningi* (Fig 5a and 5b).

**Table 2:** Relative growth of selected structures in males and females of *Pontonia manningi*. The regression equations, correlation coefficients (adjusted for d.f.), standard errors of the slopes (SE_s_), and the allometric status of each studied variable are shown (CL, PL, and AL = carapace length, length of the propodus of the major cheliped, and length of the second abdominal pleuron, respectively). ANCOVAs were used to test for differences in PL and AL between males and females. See text for details.

Sex	*y*	*x*	Regression	*r*^*2*^	SE_s_	*t*_s_	*P*	Allometry	Sexual Dimorphism
Males	PL	CL	y = 1.1481x – 0.08181	0.46	0.188	0.787	0.217	0	M > F
Females	PL	CL	y = 0.3841x + 0.1754	0.15	0.142	-4.330	<0.001	-	
Males	AL	CL	y = 0.8776x – 0.5247	0.20	0.210	-0.581	0.282	0	M < F
Females	AL	CL	y = 1.3288x – 0.5578	0.63	0.154	2.134	0.019	+	

A positive correlation was detected between CL and the length of the propodus of the major cheliped in shrimp of both sexes, as well as between CL and the width of the pleuron of the second abdominal segment in both sexes (Fig 5c). In males, the growth of the major cheliped was isometric with respect to body size; the slope of the relationship between male CL and major cheliped length did not differ significantly from unity (b = 1.15, *P* = 0.217). In females, the propodus of the major cheliped presented negative allometry; the slope of the relationship between female CL and propodus length was significantly smaller than unity (b = 0.38, *P* < 0.001). An analysis of covariance (ANCOVA) indicated a significant effect of sex (*F =* 38.38, d.f. = 1, 85, *P* < 0.0001) and CL (*F =* 63.8474, d.f. = 1, 85, *P* < 0.0001) in propodus length and the interaction term of this analysis was significant (*F* = 9.54, d.f. = 1, 85, *P =* 0.0027). Therefore, the absolute size of the cheliped and the growth rate of this structure were greater in males than in females of *P*. *manningi* (Fig 5a and 5c).

In males, the growth of the pleuron of the second abdominal segment was isometric with respect to body size (b = 0.88, *P* = 0.282), whereas in females, the same structure presented positive allometry (b = 1.33, *P* = 0.019). An ANCOVA indicated a significant effect of sex in pleuron length (*F* = 78.51, d.f. = 1, 88, *P* < 0.0001). The ANCOVA also detected an effect of CL in pleuron length (*F* = 70.01, d.f. = 1, 88, *P* < 0.0001), and the interaction term was not significant (*F* = 2.93, d.f. = 1, 88, *P* = 0.0906). Thus, the absolute size of the second abdominal pleuron, but not the growth rate of this structure, was greater in females than in males of *P*. *manningi* (Fig 5a and 5d).

## Discussion

We hypothesized that the hyper-symbiotic shrimp *Pontonia manningi* was socially monogamous, and thus, we expected that [i] the population distribution of this shrimp in *Pteria colymbus* was non-random with paired shrimp found more often than expected by chance alone, and [ii] the sex distribution of shrimp in pairs was non-random with male-female pairs being found more often than expected by chance alone. Our results agree with the two expectations above: nearly 70% of the sampled oysters harbouring shrimp were inhabited by pairs and paired shrimp were found with a frequency greater than expected by chance alone. Furthermore, shrimp found in pairs were, invariably, heterosexual. The results above also agree with that previously reported for other socially monogamous symbiotic crustaceans, in which, heterosexual pairs are found in host individuals more frequently than expected by chance alone (e.g., *Pontonia margarita* [13]; *Pontonia sp*.[39]; *Pontonia mexicana* [19]; *Planes major* [14]).

If *P*. *manningi* was socially monogamous, we also expected that [iii] males would pair with females regardless of their reproductive state. In agreement with [iii] above, males of *P*. *manningi* shared host individuals with brooding and non-brooding females, and, when brooding embryos, carrying different egg developmental stages. This observation also agrees with that reported for the congeneric shrimp *P*. *margarita*, a symbiotic species also exhibiting a monogamous mating regime [13]. If males of *P*. *manningi* were instead abandoning females soon after mating, the observed male-female association pattern would be difficult to explain. For instance, in promiscuous symbiotic and free-living species, in which the association between the sexes is temporal (heterosexual pairing is short term and males abandon females soon after mating), males are found with females close to molting and spawning a new batch of unfertilized eggs (with late stage or no embryos but mature ovaries) more frequently than expected by chance alone [40–42]. This latter pattern contrasts with the one observed in *P*. *manningi* and other monogamous crustaceans [13, 39, 43].

Overall, we interpret the information above as representing strong evidence that *P*. *manningi* actively choose to share host individuals with members of the opposite sex [rather than to live solitarily or in aggregations]. Therefore, the mating system of *P*. *manningi* can be classified as monogamy, with males and females spending time together and inhabit host individuals for long periods of time; one entire or several reproductive cycles. Nonetheless, we discuss below various other characteristics of the association between *P*. *manningi* and *Pteria colymbus* that further suggest that males might be engaging in promiscuous mating tactics, at least to some extent, in the studied species.

If *Pontonia manningi* was monogamous, we also expected [iv] male-female pairs to display size-assortative pairing and [v] shrimp body size would be positively correlated with host body size. Our results agree, but only partially, with the expectations above, and thus, with the notion of monogamy in *P*. *manningi*. At first glance, the statistically significant correlations between shrimp and host body size and between the body sizes of paired males and females argue in favour of long term male-female pairing. The conditions determining size-assortative heterosexual pairing and size-assortative shrimp-host association in symbiotic species are not completely understood. However, growth restrictions imposed by host individuals over long-term resident symbiotic organisms is typically invoked to explain such host-shrimp and male-female shrimp body size relationships ([13] and references therein). Importantly, in symbiotic species exhibiting long term monogamy, the body size relationships above are much tighter than those herein observed for *P*. *manningi*. For instance, in the symbiotic and monogamous congeneric shrimp *P*. *margarita* and in other symbiotic and socially monogamous species, the crab *Pinnixa transversalis*, male body size explains 77.6% and 63.8% of variation in female body size, respectively [29, 13]. In the monogamous species above, a positive correlation between host and symbiotic guest body size is also observed [29,13]. By contrast, only ~24% of variation in female body size was explained by male body size in *Pontonia manningi* and only ~24% and ~26% of the variation in male and female shrimp body size, respectively, was explained by host body size. This weak size-assortative shrimp pairing and loose relationship between host and shrimp body size in *P*. *manningi* suggest that the association between shrimp and host individuals and between males and females forming pairs might not necessarily be temporally stable and males do not share the same host individual for long periods of time. Instead, male (and/or female) shrimp might be shifting among host individuals and changing sexual partners, at least to some extent, as reported for other symbiotic species in which no or a weak correlation between paired male and female body size has been reported [44–45]. For instance, in *Allopetrolisthes spinifrons*, an anemone-associated porcelain crab, males move rather frequently among host individuals [45]. In this crab, the relationship between host and crab body size is negligible [46]. Lastly, at present, we cannot discard alternative mechanisms to male host-switching in determining the weak size-assortative shrimp pairing and loose relationship between host and shrimp body size in *P*. *manningi*. For instance, differential sex-specific survival rates might well explain our observation of pairs comprising partners of different sizes/ages.

Importantly, that several of the females found living solitary in oysters were brooding eggs in the studied populations further suggests that, at least to some extent, males of *P*. *manningi* are roaming around, switching among hosts in search of mating partners. In caridean shrimps, including *P*. *manningi*, females do not store sperm and need to be inseminated short after molting to fertilize a new batch of eggs [47]. Thus, if males of the studied species were staying together with their female partners in the same host individual for long periods of time, this observation would be difficult to explain. Indeed, in the monogamous symbiotic shrimp *Paranchistus pycnodontae*, females are occasionally found living solitarily in their host individuals. Nonetheless, invariably, these solitary females do not brood embryos [43]. This pattern contrasts with the one observed in *Pontonia manningi*.

A final argument suggesting that monogamy might be 'relaxed' in *P*. *manningi* is the pattern of sexual dimorphism herein observed. If *P*. *manningi* was socially monogamous, we also expected this species to display little to no sexual dimorphism in body size and weaponry (e.g., chelipeds used for intra-sexual aggression) [11–12, 48]. The observed pattern of sexual dimorphism in *P*. *manningi* agrees, but only partially, with the prediction above. On one hand, in agreement to that reported for other monogamous shrimps (e.g., *P*. *margarita* [13]; *P*. sp. [39]; *Paranchistus pycnodontae* [43]), males of *Pontonia manningi* were, on average, slightly smaller than females. Selection for large body sizes in males is expected to be relaxed in *P*. *manningi* given the low intensity of sexual selection (i.e. infrequent competition among males) characteristic of monogamous mating regimes [11, 13, 15]. Also, the major cheliped of males did not exhibit positive allometry, further arguing in favor of monogamy in *P*. *manningi*. In crustaceans where competition among males for receptive females is severe [11], males exhibit fighting appendages (i.e., chelipeds in shrimp) that exhibit positive allometric growth. This pattern contrasts with the one observed in *P*. *manningi*.

On the other hand, although the major cheliped of *P*. *manningi* did not exhibit positive allometry, males had a major cheliped larger than that of females at any given body size and the allometric scaling (relative growth rate) of this structure was greater than that of females, in contrast to that observed in various other monogamous species, including the congeneric shrimp *P*. *margarita*, in which males and females exhibit chelipeds of similar size and experience similar allometric scaling at any given body size [13]. The observed sex-specific differences in resource allocation to cheliped biomass in *P*. *manningi* disagrees with prediction of low sexual dimorphism in terms of weaponry in monogamous species [11, 48] and suggests that males are competing, at least to some extent, for receptive females via overt aggression. In order to compete, males must roam among host individuals in search of mating partners, in agreement with our observations on solitary females brooding embryos (see above). If males do switch among hosts, when resident and intruder males meet, cheliped size possibly determines the winner of the agonistic interaction and access to the receptive females [49–50]. Overall, the observed pattern of sexual dimorphism and allometric scaling of structures used as weapons suggest that competition among shrimp (and thus, host-switching) is not necessarily rare, which supports the idea that monogamy in *P*. *manningi* is not necessarily long term.

Overall, our results suggest that the body size and allocation to weaponry (i.e., cheliped size) in males of *P*. *manningi* represent an evolutionary compromise between the optimal body size (and cheliped size) for swapping among hosts while using little energy, and the optimal body size for successful intra-sex competition for females after they are found. Small body size in males might be favored if it increases agility and encounter rate with potential mating partners. In turn, large weaponry in males but not in females might be adaptive if it increases reproductive chances once receptive females have been found [35, 48]. There is a clear need for improving our understanding of the forces driving sexual dimorphism with respect to body size and weapons (chelipeds) in resource specialists.

Altogether, the above information argues in favour of monogamy in the hyper-symbiotic shrimp *P*. *manningi*, and support predictions at the core of mating systems theory [11, 15]. Further testing of particular predictions to demonstrate monogamy in symbiotic species additionally allowed us to reveal a more complex scenario (than strict long-term monogamy) that depicts a primarily monogamous shrimp, in which males likely exhibit, to some extent, a promiscuous behavior, occasionally roaming among host individuals in search of sexual partners.

What conditions might allow some moderate degree of promiscuity in this primarily monogamous hyper-symbiotic shrimp? Social monogamy in *P*. *manningi* was predicted to be a function of high risk of predation away from hosts, host scarcity and small relative host size (see Fig 1). Our field data agree with the ideas above. Supporting the idea that the risk of predation away from hosts is high, many omnivorous/predatory fishes, known to prey upon small crustaceans [24], were observed during sampling. Also, the prevalence of winged oysters in soft coral hosts was low (> 22%). The above implies that shrimps shifting among host individuals in search of sexual partners might travel tens to hundreds of meters (and thousands of times their own body length) before finding another winged oyster that might not harbour potential sexual partners (prevalence of *P*. *manningi* on winged oysters was < 40% at the different sampling sites). We believe that the conditions allowing some degree of promiscuity in this primarily monogamous hyper-symbiotic shrimp have to do with the local population distribution of the host *Pteria colymbus*. Although winged oysters were often found solitarily in ~56% of the infected soft corals, paired oysters or groups comprising 2–7 individuals were found in the other ~44% of the soft corals harbouring *P*. *colymbus*. Physical proximity among winged oysters in some but not all soft coral colonies might determine that the benefits of host-switching by male shrimp (e.g., when searching for extra-pair mating opportunities) might overcome the costs associated with the same promiscuous behavior in some but not all soft coral colonies. These costs might include, but are not limited to, increased risk of predation when away from oysters and when re-establishing the symbiotic association with host individuals that have been previously abandoned. For instance, in another crustacean endosymbiotic with bivalves, the pea-crab *Pinnotheres novaezelandiae*, dead individuals have been found trapped in between the valves of their clam hosts [51]. Mark and recapture experiments in the field should improve our knowledge of the conditions favouring the frequency with which shrimp leave their hosts in search of extra-pair mating opportunities. However, conducting such experiments might be difficult due to the cryptic habitat of the studied shrimp and the remote location of the study sites.

We have shown that the hyper-symbiotic shrimp *Pontonia manningi* exhibits a primarily monogamous mating system but in which males exhibit, to a certain degree, a promiscuous behavior. This mating system is similar to that previously reported for *Alpheus armatus*, an ectosymbiotic shrimp also found in male-female pairs in the sea anemone *Bartholomea annulata*, but in which some males do occasionally switch among host individuals in search of extra-pair copulations [2, 52]. The mating system of only two other species in the genus *Pontonia* has been studied in detail. One of them, *P*. *margarita*, inhabiting the pearl oyster *Pinctada mazatlanica* in the central eastern Pacific, appears to be strictly monogamous [13] while the second species *P*. *mexicana*, inhabiting the pen shell *Pinna carnea* in the southeastern Caribbean, appears to live in pairs too but is highly promiscuous [19]. Species of *Pontonia* inhabit host species that differ widely in terms of biology and ecology (e.g., various bivalves, gastropods, tunicates [23]). We propose that monophyletic clades of symbiotic species (e.g., *Pontonia*) exhibiting disparity in terms of host ecology represent a model group to understand the evolution of mating systems and male mating tactics in resource-specialist species.
